# Living With Interstitial Cystitis: A Qualitative Study of Women’s Experiences

**DOI:** 10.1177/01939459251387205

**Published:** 2025-11-06

**Authors:** Camilla Olaussen, Trine-Lise Jansen

**Affiliations:** 1 Lovisenberg diakonale høgskole [Lovisenberg Diaconal University College], Oslo, Norway

**Keywords:** interstitial cystitis, bladder pain syndrome, lived experience, written accounts, women’s health

## Abstract

**Background:**

Interstitial cystitis is a complex, highly challenging chronic inflammatory condition that is most predominant in women. It is characterised by persistent pain in the bladder and pelvis area and an urgent and frequent need to urinate during the day and at night. Despite the chronic and burdensome nature of interstitial cystitis, the lived experiences of women suffering from it have been inadequately explored.

**Objective:**

We sought to acquire in-depth knowledge and insights into the experiences of Norwegian women living with interstitial cystitis.

**Methods:**

A qualitative approach was used with an exploratory descriptive design, using the participants’ own written stories of living with interstitial cystitis. The data were analysed using a reflective thematic analytic method.

**Results:**

Three main themes came to the fore: (1) interstitial cystitis impacts every aspect of life, (2) struggling to be believed and heard, and (3) struggling to access personalised treatment for symptom alleviation.

**Conclusions:**

Interstitial cystitis is a complex condition that negatively impacts women’s physical, mental, and sexual health. Women with interstitial cystitis may face disbelief and medical gaslighting compounded by a lack of awareness about the condition, which hinders their access to tailored treatment and care. The findings emphasise the need for competence and empathy among healthcare professionals and underscore the importance of an increased focus on conditions predominantly affecting women.

## Introduction

Interstitial cystitis (IC), also referred to as bladder pain syndrome, is a complex, highly challenging chronic inflammatory condition with a poorly understood aetiology. It has the characteristics of persistent pain in the bladder and pelvis area that often originates from the urethra, vagina, or rectum and an urgent and frequent need to urinate during the day and at night.^1,2^ Exacerbated symptoms (flares) are common among individuals suffering from IC and involve an increase in pelvic, bladder, and often genital pain as well as urologic symptoms.^3,4^

Prior to the mid-1980s, IC was considered a rare psychosomatic disorder affecting post-menopausal women,^5^ but in 1986, it was acknowledged as a legitimate disease. The average age of onset is now 40 years and, although less common, the condition can also affect men, children, and adolescents.^5^ Patnaik et al suggested a prevalence of 45 per 100 000 women compared to 8 per 100 000 men^2^; hence, IC is most predominant in women. However, it is widely underdiagnosed, resulting in various and broad prevalence estimates.^6^

The cause of IC remains unclear. However, research has identified several possible triggers that may lead to the onset of symptoms, such as physical trauma, bacterial infections, over-distension of the bladder, autoimmune disorders, and inflammation and hypersensitivity of the pelvic nerves._7,8_ IC is also associated with other systemic pain syndromes and somatic disorders, such as irritable bowel syndrome, chronic fatigue syndrome, and fibromyalgia.^9^ It currently has no cure, and at present, there is no consensus regarding its management, a recommended therapy, or the treatment period required.^10^ Different treatments exist, such as patient education, dietary advice, stress relief, oral pharmacological treatments, intravesical installations, botulinum toxin A injections, and radical surgery for those with severe, unremitting IC.^9,11,12^ Patients with IC may need to try several treatments in combination before some relief is felt, since no single treatment has been found effective for acceptable symptom control.^13^ However, a recent study demonstrated that IC treatment efficacy is influenced by patients’ pain phenotype (ie widespread versus more localised pain).^14^ The study indicate that phenotype-directed treatment may improve outcomes: patients with predominantly local symptoms are more likely to benefit from treatments focused on urinary and pelvic pain, while those with more widespread pain may require centrally directed treatments intended for more generalised pain.^14^

The physical symptoms of IC may be accompanied by severe psychological symptoms. IC has been identified as a predictor for anxiety, depression, and insomnia,^15^ and patients may develop suicidal thoughts.^6,16^ Women suffering from IC describe the life-altering effects of IC symptoms, such as socially isolating and withdrawing from friendships due to the perceived inability of others to understand, and avoiding sexual intimacy out of concern for exacerbating symptoms and pain, as well as their widespread social burden.^17,18^

Comprehensive and collaborative treatment and care from healthcare professionals are essential for managing the symptoms and challenges associated with IC.^9^ Pain and urologic symptoms associated with IC may interfere with sleep, work, sexuality, and overall quality of life.^16-18^ Despite the chronic and burdensome nature of IC, a thorough literature search conducted in collaboration with an expert librarian revealed that the lived experiences of women affected remain underexplored—particularly within a European context, where only a single relevant study has been identified.^15^ Cultural context may influence how illness is understood,^19^ which in turn can shape individuals’ experiences of it. Moreover, as highlighted by international initiatives,^20^ variations in healthcare systems across countries can result in disparities in the quality, accessibility, and experience of care. For instance, the United States operates a fragmented, insurance-based system, whereas Norway and many other European countries offer universal health coverage through publicly funded models.^20,21^

Given this context, the objective of the present study is to generate in-depth knowledge about the experiences of Norwegian women living with IC, thereby contributing to the limited body of qualitative research on this topic within Europe.

## Methods

### Design

This study employed a qualitative approach with an exploratory descriptive design, using the participants’ own written stories of living with IC. Given the limited previous research on the lived experiences of women suffering from IC, this design was deemed suitable for providing rich data and insights into participants’ thoughts, attitudes, and experiences.^22,23^

### Participants and Sampling

This study was conducted in Norway using a purposive sampling approach. The participants, who were women aged over 18 years diagnosed with IC, were recruited via an online member-established support forum for people with IC. Recruitment adverts, including information about the study, were posted on a wall at the support forum, with written permission from the forum administrator.

### Data Collection

The recruitment adverts included a link to a secure data collection tool developed and hosted by the University of Oslo. This tool was used to distribute the study’s web-based informed consent forms and as a platform for digitally collecting data from volunteers. After signing an informed consent form, each participant was asked to digitally write and deliver an account of their experience living with IC, choosing aspects of their story that they found important to emphasise. This method allows participants to freely express themselves in their own voices and at their own pace, which can be particularly significant when disclosing sensitive issues.^24^ Furthermore, it can enhance the authenticity and credibility of the data and reduce researcher bias.^24^ The individual characteristics of our participants, such as age, marital status, or years since diagnosed with IC were not solicited, but some chose to share them in their stories. A total of 18 individual stories were collected, which was an adequate number to secure information power, as the women who wrote their stories all possessed the specific characteristics needed to meet the aims of the study.^25^

### Data Analysis

The analysis of the written data material adhered to Braun and Clarke’s reflective thematic analytic method, following 6 phases.^26,27^ The first phase involved familiarisation with the data, with both authors reading through all the written stories several times to gain a comprehensive understanding of the stories’ content and possible themes. The second phase involved coding interesting features, followed by the third phase, which involved collating these features into potential themes, focusing on how crucial the theme was in relation to the study aim. The fourth and fifth phases involved reviewing themes and defining and naming them, respectively, with each phase conducted collaboratively by both authors. Preliminary themes were reviewed and checked “against all coded data relevant to each theme.”^28(p84)^ When defining and naming themes, we emphasised that the various themes’ labels mirrored the content in a meaningful way. Throughout the entire analytic process, we read the stories separately before discussing the data and agreeing on the results. This secured analytic rigour and helped avoid bias and researcher subjectivity.^29^ The final phase involved writing the text. Analytic credibility was obtained by presenting quotations to show the reader the participants’ own descriptions of their thoughts and experiences.

### Ethical Considerations

This study was approved by the Norwegian Agency for Shared Services in Education and Research (reference no. 229012) and by the Regional Committees for Medical and Health Research Ethics (reference no. 730904). All participants were informed in writing about the study’s content, purpose, and goals; that participation was confidential and voluntary; and that they were free to withdraw from the study at any time. The potential benefits of contributing to scientific knowledge and the foreseeable risks associated with their participation were also outlined. Each participant digitally signed an informed consent form. The collected data were stored according to the Norwegian National Committee for Research Ethics for Social Science and the Humanities’ ethical guidelines.^30,31^ When presenting quotes from the participants, each woman is given a fictitious name.

## Results

Most of the participating women had been diagnosed with IC for many years, with some over 20 years. Through the written stories, it became clear that IC is a multifaceted and extensive condition that severely affects women’s lives. Three main themes came to the fore: (1) IC impacts every aspect of life, (2) struggling to be believed and heard, and (3) struggling to gain personalised treatment for symptom alleviation.

### Interstitial Cystitis Impacts Every Aspect of Life

The participants’ stories provided detailed accounts of how IC impacted every facet of their lives. Many of the women perceived the illness as dominating; it had taken over and significantly controlled their lives. Their stories consistently highlighted symptoms such as a frequent and urgent need to urinate, severe pain, fatigue, and disrupted sleep. These symptoms hindered their normal social functioning and severely limited their social life. Ingrid shared the following about her life before having a treatment regimen that relieved her of some of her pain:

The pain from IC was all-consuming. The only thing I could do was lie on the couch and exist. . . . I couldn’t drive because of the painkillers. I barely saw my family, didn’t see friends and was almost never out of the house.

Other participants similarly described social isolation and falling out of social circles. Stella, who had experienced IC symptoms since her teenage years, described the consequences that the illness had had on her life as follows:

The disease has taken away my ability to work, travel long distances, attend concerts or do other activities that require me to sit or stand for extended periods. It has also taken away my ability to have a child of my own. I was pregnant, but since I had to stop all treatments and pain relief overnight, the pain became so unbearable that I had to undergo a medically induced abortion.

The women provided detailed descriptions of the psychological effects of IC, reporting experiences of depression and anxiety, feelings of emptiness and uncertainty, mood instability, and anger. Many participants expressed profound sadness over the ways the illness had affected their lives; for instance, one woman described the pain of being unable to support her children as she wished. Participants also articulated challenges in coping with the changes in their lives and feelings of hopelessness stemming from the absence of a cure for IC. Moreover, they detailed the impact of IC on their sexual health and intimacy. They reported experiencing pain during intercourse, disruption of their sexual lives, fear of sexual activity and intimacy, and concerns about the condition’s effects on their partners and relationships. Nora wrote, “It [IC] affects my relationship, sex life and marriage. I have much less energy than before, experience a lot of pain and get little sleep most nights.”

Additionally, several participants described how IC affected their finances due to special diets and extra expenditure on electricity bills, since exposure to cold exacerbates IC symptoms. Anna wrote: “It is extra expensive with electricity; I need to keep it at at least 25 degrees so I don’t get cold. . . . My finances go into the red every month.”

Alongside the increased expenses related to IC, the illness had had a negative impact on the employment status and income of many of the women. Most participants in the study were fully or partially disabled due to IC.

### Struggling to be Believed and Heard

For some women, the onset of the disease included recurrent urinary tract infections. For others, the IC symptoms suddenly appeared as severe pain, as recounted by Hanna: “It started like a bolt from the blue after a vacation. . . . Suddenly, sharp burning pains in the bladder and pelvic area—it felt like my bladder was shattering.” One of the participants linked her onset of IC to the traumatic experience of extreme social exclusion and bullying, while another attributed it to prolonged exposure to sexualised violence.

A recurring theme among the women was their struggle to be believed and heard when seeking help and treatment for their ailments from the healthcare system. They often found that they were given little time or opportunity to share their experiences and concerns with healthcare providers. If they managed to have a consultation, it was often very brief, providing them with limited opportunities to speak and be heard. A common thread was experiences of miscommunication during medical consultations, with ailments downplayed. This sentiment was echoed in statements such as that of Stella: “I have felt misunderstood and barely heard by doctors. I have felt that my pain is trivialised.”

In all the stories, the women experienced a lack of support and understanding from healthcare professionals, to some extent. This was often attributed to the fact that IC is invisible externally, to the stigma surrounding women’s health issues in general, or to their IC frequently coexisting with other female-related disorders given low priority among the medical community. Throughout the stories, the women recounted repeated encounters with healthcare providers, during which they felt the burden of not being taken seriously. Maria described this as follows: “It was like being in a bad movie; I couldn’t believe that a patient could be treated like that. It left me feeling uncertain, depressed, like a different person.”

Many participants experienced their symptoms and pain being attributed to psychological causes by healthcare professionals. Encounters with condescending attitudes from healthcare professionals were a common thread in the women’s stories, leaving many of them feeling as though they had been seen to be exaggerating, seeking attention, or complainers. Stella wrote:

A urologist I saw was ready to prescribe me only disposable catheters because he believed that my problem was that I was too tense to empty my bladder properly. He said he could also feel “a bit stretched” when his bladder was full, making me seem like a whiner.

One of the women recounted doctors scoffing at her experiences during consultations; others had been told that their problems and pain were imagined or that they needed to pull themselves together. Eva wrote: “I’ve argued with various healthcare professionals and GPs over the years because some psychological problems I have had in the past have been used against me, with claims that my current problems are all ‘in my head.’”

### Struggling to Gain Personalised Treatment for Symptom Alleviation

In addition to the initial struggle to be believed and heard, many of the women found it extremely burdensome to fight for treatment that could alleviate their symptoms. Birgitte wrote: “I wish that those who deny us the necessary treatment could wear a belt that gives them severe pain 24/7. I believe that it would make them understand how much pain affects our and our families’ lives.”

Several women reported independently searching for information about their symptoms. Some had even suggested IC as a potential diagnosis when undergoing medical evaluations. For most women, there had been a significant delay between the onset of symptoms, receiving a diagnosis, and the subsequent establishment of an effective treatment regimen—often spanning many years. For many, this resulted in feelings of grief, bitterness, or anger over not having received help earlier. Lise shared the following: “When I asked at the hospital how long they had been offering this treatment, one urology nurse said they had had it for over 20 years. That stung, knowing I could have received help much earlier.” Sofie said, “Looking back at my medical history, I should have been diagnosed years ago. But I wasn’t, and that’s how it is.”

The participants also highlighted significant variations in the follow-up and treatment they had been offered. They kept up with research on various treatment options, often suggesting these alternatives to their practitioners. Different treatment alternatives were proposed by different practitioners, and some women felt they had to argue and fight to try various types of treatments they knew existed. Stella recounted the following:

I had to push to try to get treatment for interstitial cystitis, and I had to explain the disease and how I identified with it. After much back and forth, I was diagnosed. This marked the beginning of a lengthy trial-and-error period with treatments. I still encounter doctors with no knowledge of the condition and meet many prejudices.

Others had felt compelled to undergo treatments they did not desire, either because of severe side effects or because the treatments had not been effective for them in the past. A common experience was rarely, if ever, seeing the same practitioner; instead, they described encountering a variety of healthcare professionals with differing opinions about the best practices to treat their symptoms. Elisabeth shared the following:

I had a young urologist who really tried everything. He would call me to check in on my progress. I’ve never experienced that with any other urologist. Unfortunately, the nice urologist left. Then I saw different ones each time. One of the new urologists insisted I try [pentosan] again, even though I hadn’t previously had good results and had suffered many side effects. But if I didn’t want to take it, they wouldn’t treat me anymore.

## Discussion

The findings provide valuable insights into how Norwegian women experience living with IC, highlighting the multifaceted nature of their challenges. It is noteworthy that these findings largely align with results from qualitative studies conducted in other countries, despite differences in healthcare systems and cultural contexts.^6,17,18,32,33^

Our results demonstrate that IC imposes significant costs on individuals and severely reduces quality of life. Consistent with several other studies, our findings indicate that women diagnosed with IC endure severe pain, poor sleep quality,^32,34,35^ and psychological symptoms such as anxiety and depression.^6,34^ Moreover, IC negatively impacts intimacy, sexual functioning, and interpersonal relationships.^6,17,36,37^ Our findings align with those of other qualitative studies, showing that women diagnosed with IC face obstacles and limitations in daily life due to their symptoms, including reduced participation in social activities, decreased work capacity and challenges with personal finances.^6,17,18,32,33^

In their written stories, the participants paid considerable attention to their experiences with the healthcare system. This focus underscores the profound impact these experiences had and highlights their perceived importance. Participants consistently emphasised the lack of symptom and illness validation, the underdiagnosis, and the inadequate treatment. These findings align with previous qualitative studies.^6,17,33^ Similarly, McKernan et al found that women suffering from IC frequently questioned healthcare providers’ competence and knowledge regarding IC.^18^ This scepticism often stems from experiencing disbelief from physicians; a lack of familiarity with IC, both inside and outside the urology speciality; and the necessity to find healthcare providers knowledgeable specifically about IC for successful treatment outcomes in both medical and mental healthcare services.^17,18^ Moreover, a lack of awareness about IC and its characteristics leads patients to undergo numerous consultations without receiving appropriate treatment. This sense of not being heard in interactions with healthcare services and failing to receive the necessary treatment can constitute a significant psychological burden.^38(p14)^

Our findings indicate that women with IC frequently encounter trivialisation and psychologisation, the latter referring to the tendency to attribute physical symptoms to psychological causes without sufficient empirical justification,^39^ in their interactions with healthcare professionals. Similar experiences, including scepticism regarding the physical nature of their condition, were documented in a UK study.^17^ In an analysis of American women’s experiences with IC, Gonzales et al identified instances of psychologisation and reports of being perceived as hypochondriacs by healthcare providers.^6^ This type of experience has clear similarities to experiences described by other women with conditions that primarily affect women. Research has suggested that diseases predominantly affecting women are often assumed to be psychosomatic disorders^40^ and that women experience disbelief and invalidation of their pain more frequently than men.^41^ Women are also subjected to psychologisation more often in healthcare settings.^42^ According to Samulowitz et al,^43^ female patients are more likely to be perceived as oversensitive, hysterical, and “timewasters.” In the current study, the participants’ experiences with the healthcare system may resemble what is described as *medical gaslighting—*a phenomenon that is more likely to occur in women than in men.^44^ Medical gaslighting refers to the experiences of individuals within the healthcare system who feel dismissed, invalidated, or subjected to bias, leading to frustration, self-doubt, and a sense of isolation.^45^ A systematic review that aimed to evaluate experiences of medical gaslighting in women found, similar to the present study, that medical gaslighting leads to delayed diagnosis, the need for self-advocacy, worsening health conditions, and trauma.^45^ Diseases that predominantly affect women also rank lower on the prestige scale; this low status may influence the time it takes to establish a diagnosis.^38(p14)^

While the participants in this study provided rich descriptions of vulnerability and powerlessness in encounters with the healthcare system, we also found that strength and perseverance were phenomena that emerged through their stories. Participants described advocating for themselves, persisting in the face of adversity, challenging healthcare practitioners, educating themselves about the disease, and proposing treatments and diagnoses. Their participation in online support forums can also be understood as a self-management strategy; online peer support has proven to be significant for women living with IC.^6,17^ These findings point to the duality in human experiences. Vulnerability, strength, and perseverance were all phenomena present in the participants’ lived experiences.

### Implications for Clinical Practice

Due to the complexity of IC, sufferers are likely to encounter healthcare professionals across various settings within the healthcare system. Our findings underscore the importance of increased attention and knowledge regarding conditions that predominantly affect women as well as the necessity for a holistic approach to patient care. This approach should acknowledge the need for multidisciplinary collaboration to effectively address the diverse symptoms and challenges posed, with an emphasis on addressing what the patient themself identifies as most bothersome. Empathy and professional understanding are crucial in fostering a supportive environment that acknowledges the chronic and burdensome nature of IC, thereby enhancing the quality of care and patient experiences. Furthermore, nurse practitioners knowledgeable about IC and working with female patients may be well positioned to identify IC symptoms early and accelerate diagnostic and treatment processes.

### Study Limitations

Participation in this study was voluntary, which means that we could not ascertain whether the lived experiences shared by the participants were representative of all women living with IC. Individual characteristics such as age, marital status, and geographical setting were not collected, although they could have provided a more comprehensive overview of the sample. It is possible that the women who chose to participate were those most affected by pervasive symptoms and/or had particularly negative experiences with the healthcare system. Additionally, the findings may be specific to the context in which the participants lived and may not apply to other social or geographical settings. Despite these limitations, we believe that the insights gained are transferable to other women living with IC, thereby contributing to reducing the current paucity of knowledge in this field.

## Conclusions

We found that IC is a complex condition that can adversely threaten women’s physical, mental, sexual, and existential health. Women living with IC may experience struggling to be believed and heard, psychologisation, and medical gaslighting. This, along with a lack of awareness and knowledge about IC and different opinions on how to manage the symptoms and challenges associated with IC, makes it difficult for women to receive tailored treatment and care from the healthcare system. Our findings underscore the importance of competence and empathy among healthcare professionals when interacting with women suffering from IC. It also highlights the importance of increased attention and knowledge regarding the conditions that predominantly affect women. The current study contributes to the limited body of qualitative research exploring the lived experiences of women diagnosed with IC. Gathering written accounts appears to be a fruitful method for investigating this topic since written accounts can reveal experiences such as psychologisation and medical gaslighting, which have been inadequately described in the existing literature.
